# The Long and Winding Road—Vestibular Efferent Anatomy in Mice

**DOI:** 10.3389/fncir.2021.751850

**Published:** 2022-01-28

**Authors:** David Lorincz, Lauren A. Poppi, Joseph C. Holt, Hannah R. Drury, Rebecca Lim, Alan M. Brichta

**Affiliations:** ^1^School of Biomedical Sciences and Pharmacy, The University of Newcastle, Callaghan, NSW, Australia; ^2^Department of Otolaryngology, University of Rochester, Rochester, NY, United States

**Keywords:** vestibular, efferents, anterograde, retrograde, mouse, cre-dependent

## Abstract

The precise functional role of the Efferent Vestibular System (EVS) is still unclear, but the auditory olivocochlear efferent system has served as a reasonable model on the effects of a cholinergic and peptidergic input on inner ear organs. However, it is important to appreciate the similarities and differences in the structure of the two efferent systems, especially within the same animal model. Here, we examine the anatomy of the mouse EVS, from its central origin in the Efferent Vestibular Nucleus (EVN) of the brainstem, to its peripheral terminations in the vestibular organs, and we compare these findings to known mouse olivocochlear anatomy. Using transgenic mouse lines and two different tracing strategies, we examine *central* and *peripheral* anatomical patterning, as well as the anatomical pathway of EVS axons as they leave the mouse brainstem. We separately tag the left and right efferent vestibular nuclei (EVN) using Cre-dependent, adeno-associated virus (AAV)-mediated expression of fluorescent reporters to map their central trajectory and their peripheral terminal fields. We couple this with Fluro-Gold retrograde labeling to quantify the proportion of ipsi- and contralaterally projecting cholinergic efferent neurons. As in some other mammals, the mouse EVN comprises one group of neurons located dorsal to the facial genu, close to the vestibular nuclei complex (VNC). There is an average of just 53 EVN neurons with rich dendritic arborizations towards the VNC. The majority of EVN neurons, 55%, project to the contralateral eighth nerve, crossing the midline rostral to the EVN, and 32% project to the ipsilateral eighth nerve. The vestibular organs, therefore, receive bilateral EVN innervation, but without the distinctive zonal innervation patterns suggested in gerbil. Similar to gerbil, however, our data also suggest that individual EVN neurons do not project bilaterally in mice. Taken together, these data provide a detailed map of EVN neurons from the brainstem to the periphery and strong anatomical support for a dominant contralateral efferent innervation in mammals.

## Introduction

**Figure 1:**
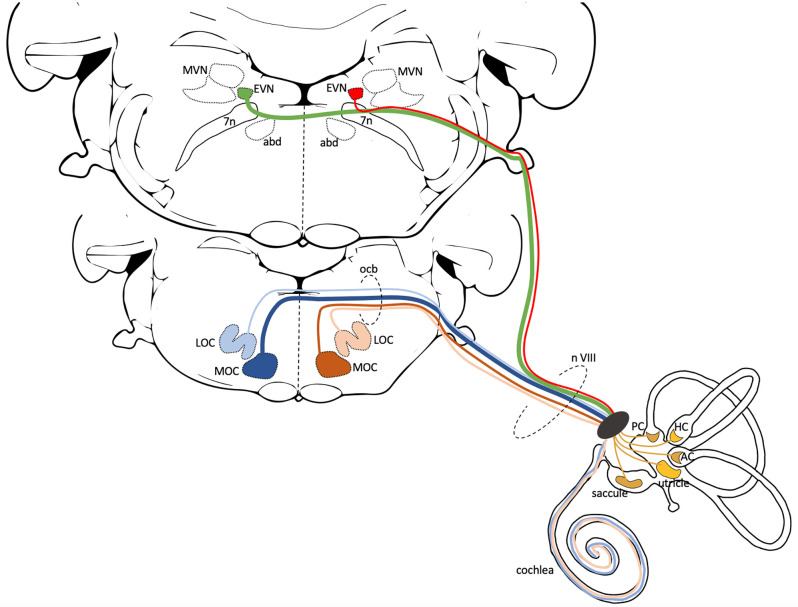
Central anatomy of the Cochlear and Vestibular Efferent System. This schematic illustrates the generic central pathways of the cochlear and vestibular efferent systems in mammals. Both efferent systems have ipsi- and contralateral projections, but here only the left side projections are illustrated. The lower brainstem slice (more rostral) shows the position of lateral and medial olivocochlear neurons (LOC, MOC). Blue lines represent contralateral, beige and brown lines represent ipsilateral efferent cochlear projections. In mice, the majority of the 475 cochlear efferent axons that innervate a cochlea are LOC neurons (~65%). Most of these LOC axons originate from the ipsilateral side (beige—99%) with only a few from the contralateral side (light blue—1%). MOC axons contribute ~35% to cochlea efferent input. Most of the MOC axons originate from the contralateral side (dark blue—75%) and the remaining from the ipsilateral side (brown—25%) (Campbell and Henson, 1988). The upper brainstem slice (more caudal) shows the position of EVN, dorsolateral to the facial nerve and genu (7n) and neighboring regions, the Medial Vestibular Nucleus (MVN), and the abducens nucleus (abd). To date, in mice, it is unclear the number or percentage of EVN cells that send ipsilateral (red line), contralateral (green line), or perhaps bilateral projections. The cochlear and vestibular efferents join the vestibulocochlear nerve (n VIII) as it exits the brainstem and travel together to the inner ear. The cochlear projections innervate the inner hair cells (LOC efferents) and outer hair cells (MOC efferents) in the organ of Corti. The efferent vestibular projections innervate Type II hair cells, afferent nerve fibers, and afferent calyx terminals in the five vestibular organs: saccule, utricle, anterior crista (AC), horizontal crista (HC), posterior crista (PC).

Two separate and distinct efferent systems transmit information from the central nervous system to peripheral inner ear organs (Figure 1). (1) The olivocochlear (OC) system comprises medial and lateral olivocochlear neurons that innervate the cochlea; and (2) efferent vestibular system (EVS) consisting of efferent vestibular nucleus (EVN) neurons that innervate the vestibular organs (Ryugo et al., 2011; Cullen and Wei, 2021). Superficially, the two systems share similarities in form and function, and therefore it has been tempting to consider them as operationally equivalent. Indeed, both auditory and vestibular efferent neurons are localized to the brainstem and they both have a similar mechanism of action, using cholinergic and peptidergic neurotransmitters to modify peripheral hair cell and primary afferent activity (Ryugo et al., 2011; Cullen and Wei, 2021). Both groups of efferent neurons are also proportionately fewer in number when compared to their afferent counterparts, and both efferent systems branch extensively to provide a significant peripheral terminal distribution within their respective inner ear organs (Brown, 2011; Holt et al., 2011). However, closer inspection reveals there are key differences that are likely to influence their function. For example, despite being in the brainstem, the location of the auditory and vestibular efferent neurons are different and therefore descending influences are likely dissimilar. OC neurons are found ventrally in the trapezoid body and the lateral superior olive (Brown, 2011), while EVN neurons are typically more dorsal, lying above the facial genu and below the vestibular nucleus complex (Holt et al., 2011). Both efferent systems receive innervation from targets of their afferent counterparts. Thus, OC neurons receive innervation from the cochlear nucleus, while EVN neurons receive innervation from the vestibular nucleus complex. To examine these differences further, however, requires a detailed anatomical understanding of both efferent systems in the same animal model. While auditory efferents have been studied broadly across many species, vestibular efferents have received much less attention. To date, the gerbil is the only rodent model for which there is detailed information on both central and peripheral vestibular efferent anatomy (Perachio and Kevetter, 1989; Purcell and Perachio, 1997). In mouse, a more common laboratory animal and for which there is the requisite detail on auditory efferent anatomy, there are only isolated studies of EVN neurons (Leijon and Magnusson, 2014; Mathews et al., 2015) and even less anatomical data on vestibular efferent central pathways or peripheral innervation patterns (Jordan et al., 2015). Therefore, our study has attempted to fill in the anatomical gaps of the mouse EVS so that it would be possible to examine both the auditory and vestibular efferent systems in the same species.

We used three transgenic mouse lines and two different tracing strategies to examine *central* and *peripheral* vestibular efferent anatomy. By using Cre-dependent, AAV-mediated expression of fluorescent reporters we were able to label the left and right EVN neurons, separately, with different colored fluorophores. The labeling included cell bodies, dendritic architecture, axonal projections, and efferent terminals in vestibular neuroepithelia. EVN dendritic arborization extended well into adjacent dorsal and medial regions including the vestibular nucleus complex. We traced their respective axonal trajectories as they exited the mouse brainstem with the vestibulocochlear nerve. In addition, we were able to follow these color-coded axons to their peripheral terminal fields in the same vestibular organs. We coupled these anterograde results with Fluro-Gold retrograde labeling to quantify the proportion of ipsi- and contralaterally projecting cholinergic efferent neurons. Our results support the absence of individual EVN neurons projecting bilaterally and confirm previous studies that identified the location of mouse EVN neurons (Leijon and Magnusson, 2014; Mathews et al., 2015). However, our results are in contrast with the peripheral innervation patterns described in the gerbil (Purcell and Perachio, 1997). This suggests there may be significant interspecies differences in the organization, and perhaps the function, of the EVS. Therefore, as suggested previously, caution should not only be exercised when extrapolating EVS results from other vertebrate classes to mammals (Holt et al., 2011), but even among members of the same order, such as rodents.

## Materials and Methods

### Bioethics Statement

All experimental procedures were approved by The University of Newcastle Animal Care and Ethics Committee prior to experiments. Adult mice (4–8 months and both sexes) from three transgenic mouse strains were used in this study. **Strain 1**, “*Chat-Cre”* (B6;129S6-*Chat^tm2(cre)Lowl^*/MwarJ; JAX #028861), is a homozygous driver mouse line expressing Cre recombinase in cholinergic neurons. **Strain 2**, “*Chat-ChR2*”, is the heterozygous line resulting from the crossing of homozygous *Chat-Cre* mice with homozygous Ai32 mice (B6.Cg-Gt(ROSA)26Sor^tm32(CAG-COP4*H134R/EYFP)Hze^/J; JAX #024109). *Chat-ChR2* mice co-express the light-sensitive channel, channelrhodopin-2, and enhanced yellow fluorescent protein (ChR2-eYFP) in cholinergic neurons. **Strain 3**, “*Chat-gCaMP6_f_*”, is the heterozygous line resulting from the crossing of homozygous Chat-Cre mice with homozygous Ai95D mice (B6J.Cg-*Gt(ROSA)26Sor^tm95.1(CAG-GCaMP6f)Hze^*/MwarJ; JAX #028865). *Chat-gCaMP6_f_* mice express the calcium indicator protein GCaMP6_f_ in cholinergic neurons. Strain 1, *Chat-Cre* mice, were used for Cre-dependent anterograde viral tracing studies and as a foundation line for *Chat-ChR2* and *Chat-GCaMP6_f_* strains. *Chat-ChR2* mice were used for general anatomical mapping of cholinergic neurons, and *Chat-GCaMP6_f_* mice were used for retrograde labeling studies. Genotyping was done in conjunction with Australian BioResources and the Garvan Institute, using the standard forward and reverse primer sequences recommended by the Jackson Laboratory for these commercially available live strains.

### Anterograde Tracing With Adeno-Associated Viruses (AAVs)

**Figure 2:**
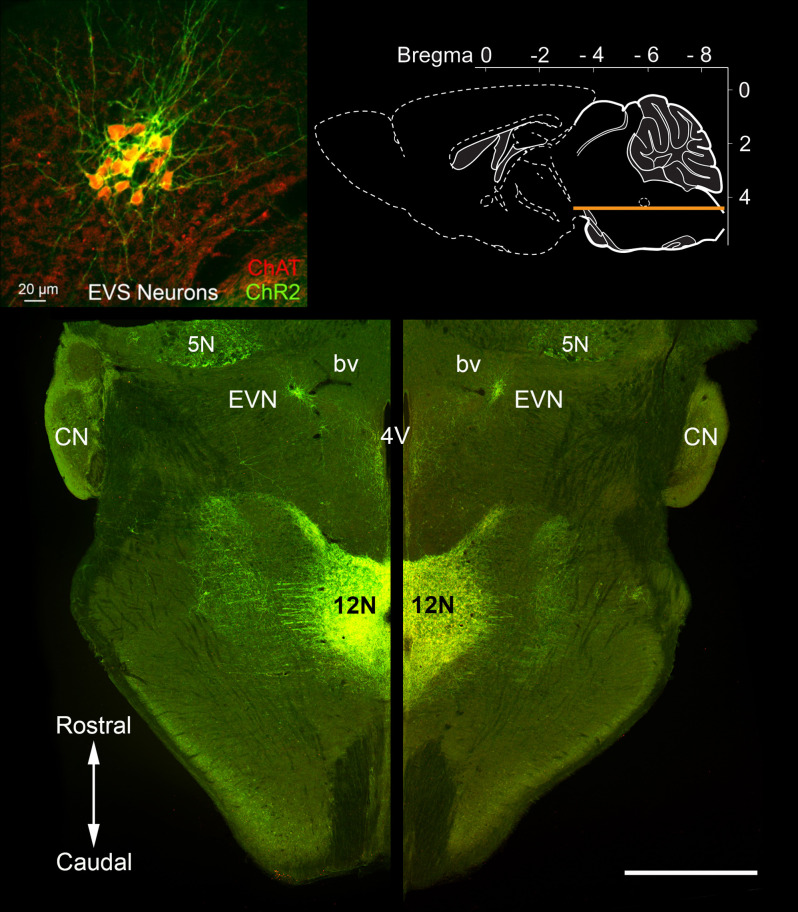
Rostrocaudal location of the Efferent Vestibular Nucleus (EVN). A horizontal section through the brainstem (see top right inset) of a *Chat-ChR2* mouse, where enhanced yellow fluorescent protein (EYFP) and the light-sensitive channels, Channelrhodopsin-2 (ChR2), are co-expressed in cholinergic neurons. The tightly packed Efferent Vestibular Nucleus (EVN) neurons, which are the source of efferent input to the vestibular periphery, are fluorescently labeled and localized at the same level as the floor of the fourth ventricle (4V). EVN neurons were confirmed as cholinergic by double-labeling with antibodies against EYFP (ChR2) and choline acetyltransferase (ChAT), the enzyme responsible for the synthesis of acetylcholine. There was a complete overlap of the two labels as shown in the top-left inset. Similar one-to-one overlap was also observed with our *Chat-gCaMP6_f_* mouse strain. A characteristic feature seen in both horizontal and transverse slices is the close association between the EVN and nearby blood vessels (bv) as can be seen on both left and right sides. CN = Cochlea Nucleus; 5N = Trigeminal nucleus; 12N Hypoglossal nucleus. Scale bar: 1 mm.

**Figure 3:**
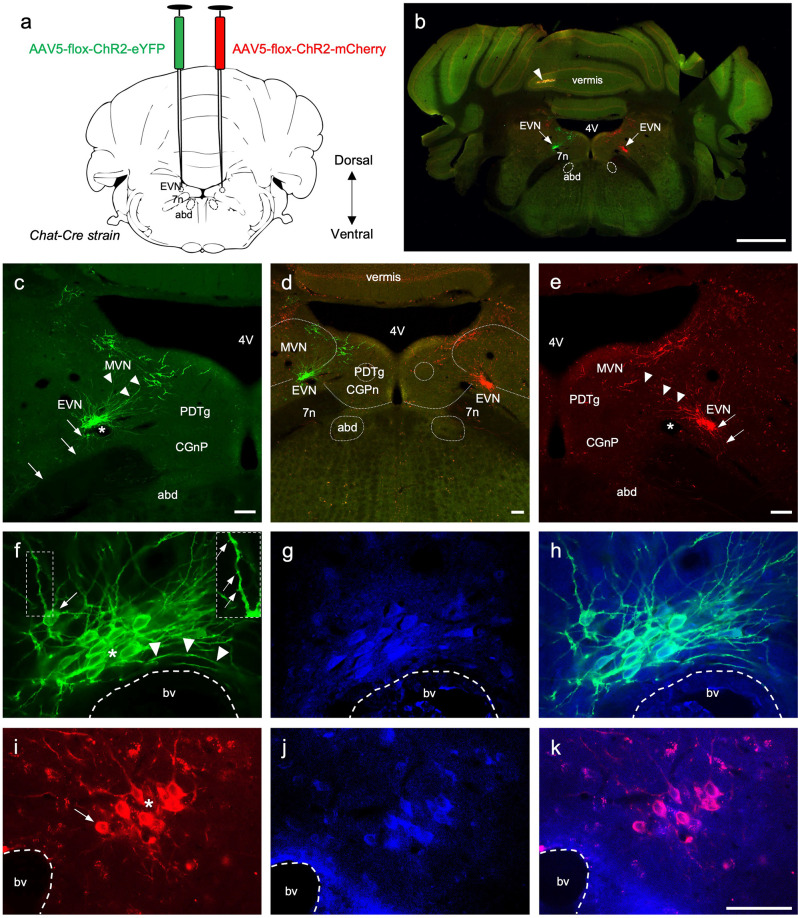
The central components of the Efferent Vestibular System. **(A)** A schematic of a transverse section showing the double intracranial virus injection experiments in *Chat-Cre* strain. AAV5-flox-ChR2-eYFP virus was injected in the left Efferent Vestibular Nucleus, EVN (green), and AAV5-flox-ChR2-mCherry virus were injected in the right EVN (red). 7n—genu of the facial nerve, abd—abducens nucleus. **(B)** Photo-merged image of brainstem transverse section at the level of the EVN following adenovirus injection. The right side of the cerebellum was purposely nicked, prior to sectioning, to help orient sections when mounting. Arrows point at the viral-induced expression of EYFP (green) on the left, and mCherry (red) on the right. 4V—4th ventricle, arrowhead—yellow fluorescent signal in the cerebellar vermis. Scale bar: 1 mm. **(C–E)** Left panel, EVN (green channel), middle panel composite image (red and green channels), and right panel EVN (red channel). Both EVNs show extensive dorsomedial branching towards neighboring brain regions, which include MVN—Medial Vestibular Nucleus, PDTg—posterodorsal tegmental nucleus, CGPn—central gray of the pons. Arrows indicate descending axonal projections; arrowheads indicate dendritic branches; asterisks show lumen of blood vessels. Fluorescent dendritic fibers were observed beyond the arrowheads (on both sides) and a subgroup of cholinergic MVN neurons which had more robust dendritic architecture and could be easily distinguished from EVN dendrites. Scale bar: 100 μm. **(F–H)** Left and middle panels, higher magnification of EVN following GFP (green) and ChAT (blue) immunohistochemistry. Right panel, a composite. Right Inset panel **(F)**: dendrite, arrows pointing to dendritic spines. Arrowheads in **(F)**—fibers curved around the blood vessel. bv—blood vessel; asterisk—tightly packed EVN cells; arrow in **(F)**—cell at the periphery of the tightly packed EVN group. **(I–K)** Right and middle panel, EVN following RFP (red) and ChAT (blue) immunohistochemistry. The right panel is the composite image. bv—blood vessel, asterisk—tightly packed EVN cells, arrow—EVN cell at the periphery of the nucleus. Scale bar **(F–K)**: 50 μm.

*Chat-Cre* mice were anesthetized using isoflurane (5% induction, 1.5–2% maintenance) and placed in a standard head-fixed mouse stereotaxic frame (David Kopf Instruments, USA). Two different adeno-associated viruses (AAVs) were injected unilaterally into the brainstem, to produce either *Cre*-dependent ChR2-mCherry (AAV5/Efla-DIO-hChR2-(H134R)-mCherry, Addgene, #20297) or *Cre*-dependant ChR2-eYFP (AAV5/Efla-DIO-hChR2-(H134R)-eYFP, Addgene, #20298) expression on opposite sides of the brainstem. In wild type mice (C57BL6) antibody labeling against ChAT protein allows for the location of cholinergic cell bodies but does not provide clear labeling that spans the dendritic and axonal fields of Efferent Vestibular Nucleus (EVN) neurons. Therefore, using ChR2, which is commonly used as a light-sensitive circuit mapping tool, here we exploit its membrane-bound properties for anatomical purposes. The dense and uniform distribution of ChR2, together with co-expressed eYFP, within the membranes of cell bodies, dendrites, and axons allowed for enhanced cytoarchitectural labeling of EVN neurons, both centrally and peripherally. AAVs generating ChR2-mCherry and ChR2-eYFP were injected into the left and right EVN, respectively. For comparison, in two cases we reversed the injection sites, mCherry virus into the right side, and eYFP into the left. No difference in label or distribution was observed. Using pulled glass micropipettes (Drummond glass, 3.5", 3000203G/X), the virus was injected intracranially (200–400 nl) using a microinjection system (Nanoinject II, Drummond SCI, USA) at a rate of 1nl/s. The coordinates of the EVN were initially determined using Mouse Brain Atlas (Paxinos and Franklin, 2001) but were later modified in response to more detailed targeting studies (Figure 2). In a typical 26 g adult mouse, AAV injections used the following co-ordinates: *X* = ± 0.7, *Y* = 5.8, *Z* = 4.5 mm from bregma, where X represents the interaural axis (or distance from the mid-line), Y the anteroposterior line (rostrocaudal) axis, and Z is the dorsoventral distance (or depth) from the bony surface of the skull at bregma. Using these co-ordinates, we specifically labeled the EVN, without interfering or labeling the large cholinergic abducens nucleus located more ventrally (Figures 3A,B). Mice were closely monitored during and after the surgeries in accordance with an institutionally approved animal ethics protocol and for optimal viral transduction were allowed to recover for up to 3 weeks.

**Figure 4:**
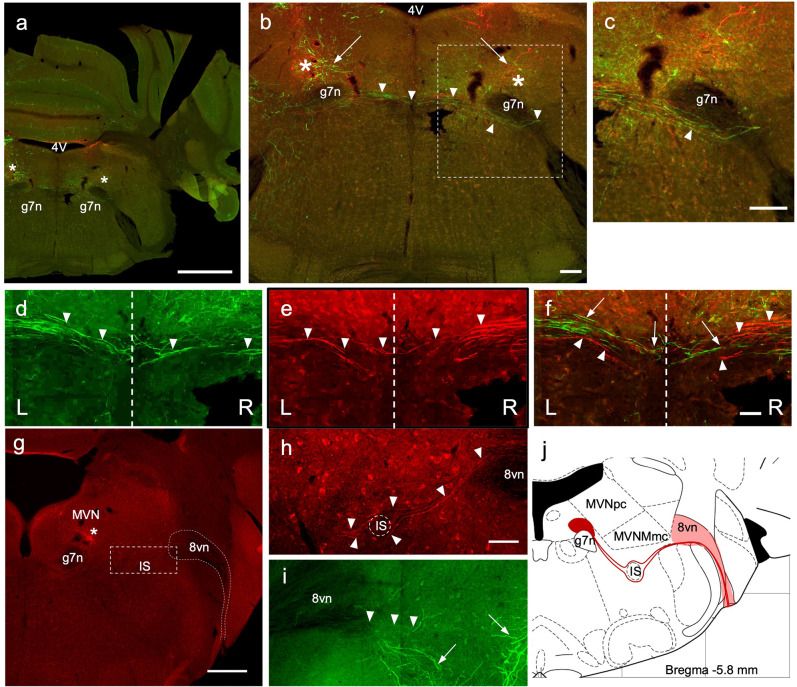
Crossing EVN fibers. **(A)** Photo-merged image of a transverse brainstem section at the level of the crossing fibers. The right-side cerebellum was nicked before sectioning for orienting. 4V—4th ventricle, 7n—genu of the facial nerve. Asterisks represent a projected area where EVN cell bodies would be in more caudal slices. Scale bar: 1 mm. **(B)** Virally induced fluorescent efferent axons crossing the midline (arrowheads). 4V—4th ventricle, arrows—level of the EVN and fluorescent branches, 7n—genu of the facial nerve. Scale bar: 100 μm. **(C)** Higher magnification of “b” dashed box, showing the trajectory of contralateral efferent axons after crossing the midline, going underneath (arrowhead) the genu of the 7th nerve (g7n) and heading towards the eighth nerve exit. **(D–F)** Green and red efferent axonal fibers, originate from the left and right EVN, respectively, and cross the midline (dashed line) of the brainstem. Arrowheads point to crossing fibers. In composite panel **(F)**. Arrows—green (left) fibers; arrowheads—red (right) fibers. Scale bar **(D–F)**: 50 μm. L = Left, R = Right. Scale bar: 100 μm. **(G–J)** Low magnification **(G)** of the right side (red) showing labeled red fluorescent EVN cell bodies (below the asterisk) located near blood vessels. Since there are fewer ipsilateral axons, they were harder to trace than contralateral axons. Nevertheless, we saw ipsilateral axons descend ventrally (**I**; green) and then re-ascend, dorsally (**H**; red). As the ipsilateral axon bundle ascends it divides around the inferior salivary nucleus **(G,H)** on its way to the vestibular root of the vestibulocochlear nerve (8vn). This tortuous and winding trajectory of ipsilateral axons is summarized in schematic **(J)**. Scale bar for panel **(G)** is 500 μm; Scale bar 50 μm for panels **(H,I)**.

**Figure 5:**
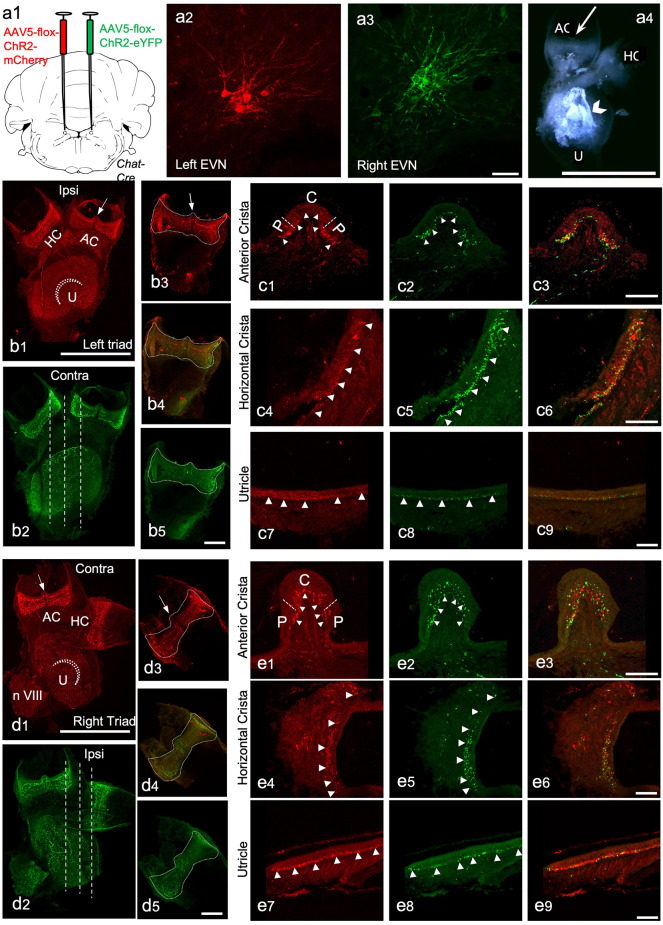
The peripheral components of the Efferent Vestibular System. We used adeno-associated viruses (AAVs) to induce the expression of red and green fluorescent marker proteins in EVN neuronal cell bodies, their axons, and terminal fields in peripheral vestibular organs. Here we show the peripheral vestibular organs. **(A)** **a1**—the injection sites of AAV5-Chr2-mCherry (left) and AAV5-Chr2-EYFP (right) anterograde viruses in the brainstem; **a2,a3**—viral labeling of left EVN by mCherry red fluorescent protein and right EVN by EYFP fluorescent protein, Scale bar: 50 μm; **a4**—dissected vestibular organs, a semi-intact triad of anterior crista (AC), horizontal crista (HC) and utricle (U), arrowhead points at the otolith crystals, Scale bar: 1 mm. **(B)** **b1,b2**—maximum projection of z-stack from the left side triad visualized using red (mCherry) and green (EYFP) channels ~3 weeks after virus injection in the brainstem. Arrow—eminentia cruciata; straight dashed line-showing the planes of sectioning; dashed curved line-striola in the utricle, HC—horizontal crista, AC—anterior crista, Scale bar: 500 μm; **b3–b5**—maximum projections of the left side posterior crista in red **(b3)** and green **(b5)** channels and the composite image **(b4)**, dashed line shows the outlines of the crista, arrow indicates eminentia cruciata, Scale bar: 100 μm. **(C)** Cross-sections of the left side anterior crista, horizontal crista, and utricle following immunohistochemistry against RFP (red) and GFP (green). **c1,c2**—anterior crista in red and green channels, dashed lines separate the central (C) and peripheral (P) parts of the crista, arrowheads point at the efferent nerve end terminals; **c3**—the composite image of the red and green channels; **c4–c6**—transverse section of the horizontal crista in red and green channels and the composite image, arrowheads point at efferent terminals; **c7–c9**—section of the utricle in red and green channels and the composite image, arrowheads point at nerve terminals. Scale bar: 50 μm. **(D)** **d1,d2**—Maximum projection from z-stacks of the right side triad in the red and green channel following virus injection in the brainstem, arrow—eminentia cruciata, straight dashed line-showing the planes of sectioning, dashed curved line—striola in the utricle, HC—horizontal crista, AC—anterior crista, Scale bar: 500 μm; **d3–d5**—maximum projections of the right side posterior crista in red **(d3)** and green **(d5)** channel and the composite image **(d4)**, dashed line outlines the crista, arrow—eminentia cruciata, Scale bar: 100 μm. **(E)** Cross sections of the right-side anterior crista, horizontal crista, and utricle after RFP (red) and GFP (green) immunohistochemistry. **e1,e2**—anterior crista in red and green channels, dashed lines separate the central (C) and peripheral (P) parts of the crista, arrowheads point at the efferent nerve end terminals; **e3**—composite image of the red and green channels; **e4–e6**—transverse section of the horizontal crista in red and green channels and the composite image, arrowheads point at nerve end terminals; **e7–e9**—section of the utricle in red and green channels and the composite image, arrowheads point at nerve terminals. Scale bar: 50 μm.

Fifteen (nine males, six females) animals were used for intracranial bilateral virus injection. The brain and vestibular tissue were processed for anatomical studies from all animals. The virus injection surgeries were successful in eight animals (six males, two females), and these were used for final anatomical validation, where the processed tissue was of good quality and fluorescent protein was strongly expressed in the brain and inner ear tissue for confocal imaging. Figures 3–5 are from three of these mice.

### Retrograde Tracing With Fluoro-Gold

*Chat-GCaMP6_f_* mice were anesthetized and placed in a stereotaxic frame, as described above for anterograde labeling experiments. For retrograde injections, we used a trans-canicular approach based on a procedure described by Guo et al. (2018). The head was rolled 45 degrees to one side allowing easier access for a post-auricular incision. The skin was reflected and the translucent temporal bone, overlying the posterior canal, was exposed, and pierced using a fine 30G needle allowing direct access to the underlying membranous posterior semicircular canal. Fluoro-Gold (FG; 4 μl; Fluorochrome, LLC; Denver, CO) was slowly injected by hand over a 5-min period using a pulled glass electrode (Drummond, 3.5", 3000203G/X) attached and sealed with dental wax to a Hamilton syringe. After injection, the hole in the bony semicircular canal was plugged with dried muscle tissue, held in place with adhesive (Vetbond, 3M, No.14690), and the skin incision closed with sutures. The mice were regularly monitored during the recovery and post-operative period that ranged from 3 to 7 days.

**Figure 6:**
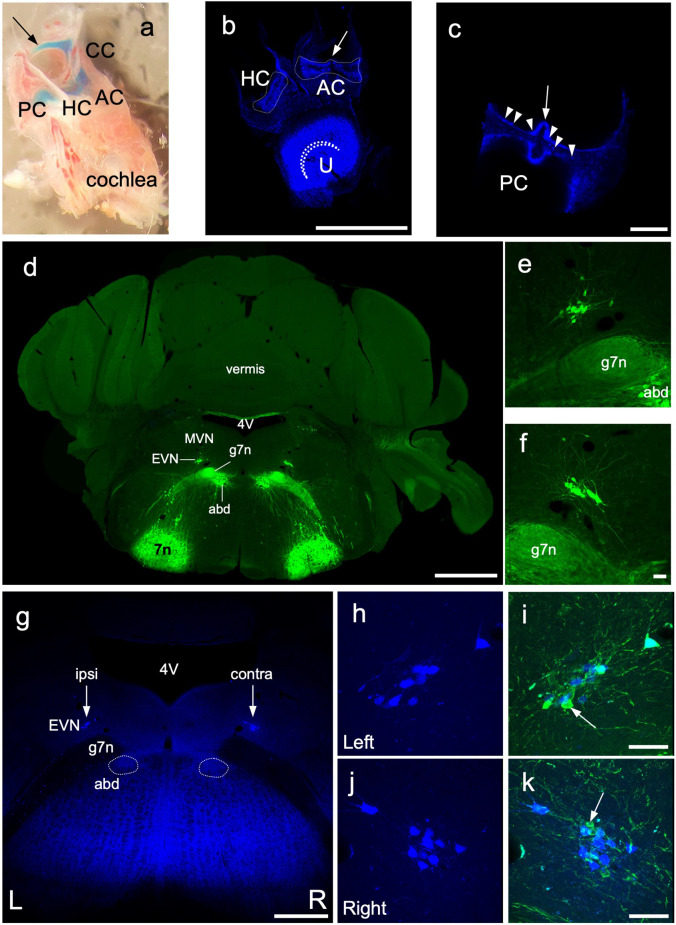
Retrograde labeling of central EVN neurons. Fluoro Gold fluorescent retrograde tracer was injected unilaterally in the inner ear through the posterior semicircular canal and the efferent neurons were traced back retrogradely in the EVN on both sides. **(A)** Dissected bony inner ear following Fast Green dye injection through the posterior semicircular canal, arrow—injection site, PC, posterior semicircular canal, CC, common crus; AC, anterior crista; HC, horizontal crista. **(B,C)** Maximum projection image from z-stacks of the triad and posterior crista following dissections of a Fluoro Gold injected inner ear, dashed line outlines the vestibular cristae. curved dashed line—utricular striola, AC, anterior crista; HC, horizontal crista; U, utricle; arrow—eminentia cruciata; arrowheads—fluorescent neuroepithelium. Scale bar **(B)**: 500 μm, Scale bar **(C)**: 100 μm. **(D)** Photo-merged image of transgenic *Chat-gCaMP6_f_* mouse used for retrograde tracing and EVN cell counting. The ChAT dependent GCaMP6_fast_expression was visualized by GFP immunolabeling in green. 4V—4th ventricle, MVN—medial vestibular nucleus, EVN—Efferent Vestibular Nucleus, abd—abducens nucleus, g7n—genu of the facial nerve, 7n—facial nucleus. Scale bar: 1 mm. **(E,F)** Left and Right side EVN nuclei under higher magnification. abd—abducens nucleus, 7n—genu of the facial nerve. Scale bar: 50 μm. **(G)** Fluoro Gold (FG) labeling of the EVN. 4V—4th ventricle, EVN—Efferent Vestibular Nucleus, arrows—position of the EVN, abd—abducens nucleus, 7n—genu of the facial nerve. Scale bar: 500 μm. **(H,I)** FG labeling in the left side EVN and the composite image of GFP and FG labeling. Arrow points to GFP but not FG-positive EVN neuron. Scale bar: 50 μm. **(J,K)** FG labeling in the right side EVN and the composite image of GFP and FG labeling. Arrow points to GFP-positive, FG-negative EVN neuron. Scale bar: 50 μm.

From the *Chat-GCaMP6_f_* strain four animals (all males) were used for the quantification of retrograde tracing. Two females were also injected in the inner ear, however, labeling was unsuccessful in their case. Images from two mice are shown on Figure 6 as representative images for retrograde Fluoro Gold labeling.

### Tissue Preparation and Immunofluorescent Labeling

After the respective post-surgical recovery times, mice were deeply anesthetized with an intraperitoneal cocktail of ketamine (100 mg.kg^−1^) and xylazine (0.01 ml/g). Once deeply anesthetized, mice were transcardially perfused with heparinized saline, followed by 4% paraformaldehyde in 0.1 M phosphate-buffered saline (PFA). The brainstem and inner ears were isolated and post-fixed for 3 h in fresh PFA. After rinsing in phosphate-buffered saline (PBS), the inner ears were isolated under a dissecting stereo microscope (Leica, M165 FC), the triad of the anterior and horizontal ampullae, and the utricle were gently freed from the surrounding bone and the overlying ampullary and utricular membrane was detached using a fine scissor to expose the vestibular neuroepithelia and to remove otolith crystals from the utricles. Isolated individual posterior cristae were processed similarly. Following the inner ear dissections, the vestibular organs were mounted on a concave microscope slide, cover-slipped using 50% glycerol/PBS solution, and were imaged as wholemounts. After imaging, triads were cryoprotected in 30% sucrose/PBS solution overnight, prior to sectioning at 30–50 μm using a Leica CM1950 cryostat. For anterograde labeling of fine processes in brainstem slices (dendrites, axons) and peripheral vestibular organ slices (axon terminal fields), we used immunofluorescent labeling to enhance red fluorescent protein (mCherry) and eYFP signal. Triads were embedded in OTC mounting media and were sectioned (30 μm) in a cryostat.

For retrograde FG labeling experiments, EVN cells were visualized in the *Chat-gCaMP6_f_* mice, through immunolabeling amplification of GFP signal. In a subset of virally transfected EVN neurons we also immunolabeled ChAT-positive cell bodies in brainstem slices and efferent terminals in the peripheral vestibular organs.

Brainstem and vestibular organ sections were incubated in primary antibodies (anti-RFP, 1:200, Chromotek; anti-GFP, 1:200, Abcam; and anti-ChAT, 1:150, Merck) for 2 days. After primary antibody incubation, the tissue was washed three times in 0.1 M PBS for 10 min and then incubated in secondary antibodies (Alexa 405, 1:50; Alexa 488, 1:200; and Alexa 594, 1:200, Abcam) for 2 h then washed in 0.1 M PBS, mounted and cover-slipped in 50% glycerol PBS mounting solution.

### Microscopy and Image Processing

EVN neurons in brainstem sections and their efferent terminals in vestibular organs were imaged using a Nikon C1 confocal microscope with 20×, 40×, and 60× objectives. To count the number of GFP- and FG-labeled EVN neurons, the brainstem slices were imaged with a z-stack step size of 0.7 μm. To visualize the efferent terminal fields z-stack images were collected at step size 0.65 μm. Images were processed and reconstructed as maximum intensity projections or 3D reconstructions using ImageJ (NIH Image) and FluoRender (Scientific Computing and Imaging Institute, University of Utah) software.

### Statistics

GraphPad Prism software (version 9.2.0, GraphPad Software, Inc, CA, USA) was used for statistical analysis. When analyzing the frequency of GFP-positive ipsilateral and contralateral EVN neurons, with and without Fluorogold (FG) labeling, we used 2 × 2 contingency tables in which the G-Statistic test of independence was computed with a Williams’ correction for type I errors (Sokal and Rohlf, 1981). Statistical significance was set at *p* < 0.05. A significant result indicated that the distribution of FG-positive cells constituted a separate population from those that were FG-negative.

## Results

### The Central Efferent Vestibular System (EVS)

We used a *Chat-ChR2* mouse line (*n* = 5; two males and three females) to explore the precise location and co-ordinates of the EVN nucleus. Horizontal brainstem sections like that shown in Figure 2, provided the rostrocaudal location, and coronal sections provided the dorsoventral location of the EVN (Figure 3). Expression of eYFP in *Chat-ChR2* animals showed a one-to-one colocalization with ChAT labeling (inset, Figure 2).

To target the EVN specifically, we intracranially injected Cre-dependant AAVs into Chat*-Cre* mice. This resulted in strong endogenous expression of eYFP and mCherry in the left and the right EVN, respectively (Figures 3A,B arrows), and could be clearly visualized using confocal microscopy without amplification with antibodies (Figure 3B). Using this approach, we were able to avoid off-target labeling of nearby cholinergic populations such as the abducens nucleus (abd) and the olivocochlear system. In two cases, we reversed the injection sites, such that the left EVN was injected with the ChR2-mCherry virus and the right EVN was injected with the ChR2-eYFP virus (Figure 5a1). Both viruses strongly expressed the fluorescent marker proteins in the brain and vestibular tissue, although in most cases the eYFP signal was more intense than that of mCherry under our confocal microscopy conditions. Both viruses effectively labeled EVN cell bodies, their primary and distal dendrites, their axon fibers, and peripheral terminals (Figures 3–5).

Nearly all eYFP- and mCherry-labeled cell bodies were contained tightly within a single EVN. Sporadically, some labeled cell bodies were found above the injection site in the Medial Vestibular Nucleus (MVN) close to the ventricular border (Figures 3C–E). The dendrites of these MVN labeled neurons could be easily distinguished from those of the EVN neurons as they were thicker and located well above (dorsal to) the EVN. Our results suggested only one EVN population or cluster on each side of the brainstem in mice and this was confirmed by our retrograde labeling results (see below). The EVN nucleus was located dorsolateral to the genu of the facial nerve bundle (g7n), approximately 0.7 mm from the midline, and −5.8 mm caudal from Bregma (Paxinos and Franklin, 2001). Distinctive features of the EVN are its relatively small size compared to the abducens nucleus, dense neuronal packing, and rich dendritic projections. The majority of EVN cell bodies formed the “core” of the EVN nucleus and overlapped with one another (Figures 3F,I, asterisk). However, some labeled EVN neurons were found further away from the dense core (Figures 3F,I, arrow), although still within 50 μm of the core cluster.

EVN neurons exhibit extensive dendritic branching, mainly dorsal and dorsomedial, infiltrating the ventral parts of the neighboring MVN (Figures 3C,E; arrowheads). In addition, dendritic fibers were also found reaching towards the *posterodorsal tegmental nucleus* (*PDTg*) and the *central gray of the pons* (*CGnP*; Figure 3D).

EVN axon fibers, thinner than primary dendrites, were also visualized. Originating from the ventral side of the EVN (Figures 3C,E, arrows), axon fibers bound for the ipsilateral and inner ear projected ventrolaterally, in the direction of the root of the eighth cranial (vestibulocochlear) nerve. However, axon fibers bound for the contralateral inner ear first projected rostrally before crossing the midline, and then returned caudally to rejoin the ventrolateral projecting fibers originating from the EVN on that side. Unexpectedly, yellow (red, and green overlap) labeling was found in two animals in a constrained area of the cerebellar vermis (Figure 3B, arrowhead, Supplementary Figures 1A–C).

In some cases where we needed to visualize finer structures, we used immunofluorescent labeling (GFP, RFP) to enhance virally expressed green and red fluorescent signals in labeled EVN neurons for better resolution of cytoarchitecture. EVN neurons and their projections were delineated in clear detail (Figures 3F,I), and in many cases, we were able to visualize dendritic spines (see Figure 3F inset). In addition, antibody labeling confirmed the simultaneous presence of ChAT and therefore the cholinergic identity of these neurons (Figures 3F–K). In all cases, where EVN neurons were virally tagged with eYFP or mCherry, they also labeled positive for ChAT (blue, Figures 3G,J)), further demonstrating the one-to-one labeling specificity (Figures 3H,K).

It should be noted that the EVN was always found close to a characteristic local blood vessel (bv, Figures 3F–K), which often impacted neuronal cytoarchitecture as fibers curved around the vessel (Figure 3F, arrowheads). In one case, the blood vessel separated EVN neurons.

### EVN Contralateral Axons Cross the Midline Rostral to Their Cell Bodies

As described above, to view labeled processes, particularly the axonal fibers, we amplified the eYFP and mCherry signal using primary antibodies against GFP and RFP, respectively. We were able to visualize the axons originating from the EVN and identify the precise location where the axonal bundle crossed the midline of the brainstem and projected toward the contralateral vestibulocochlear nerve (Figure 4). The level of the crossing fibers (decussation) was rostral to the location of EVN cell bodies by approximately 100 μm (−5.6 mm from Bregma). Note, at this level (Figures 4B,C) there were only green and red distal dendrites of EVN neurons on the left side and right side, respectively (Figure 4B, left and right arrows).

A green axonal fiber bundle originating from the left side EVN (Figure 4D, arrowheads; Figure 4F arrows) and red fibers from the right side EVN (Figures 4E,F, arrowheads) crossed the midline of the brainstem at the same level. Two features can be observed from these contralaterally projecting axon bundles. (1) Following their decussation, the bundles travel ventral to the genu of the facial nerve (Figure 4C, arrowhead); and (2) the contralateral axonal bundle appeared to be larger relative to the number of ipsilateral axons.

### EVN Ipsilateral Axons Stay at the Level of Their Cell Bodies

Although ipsilateral EVN axons exhibited endogenous fluorescence following injection of Cre-dependent AAVs, amplification *via* immunofluorescent labeling allowed for better visualization of finer structures (Figures 4G–I). EVN fibers projecting to the ipsilateral inner ear appeared fewer in number and less densely packed than those projecting to the contralateral inner ear. The trajectory of ipsilateral axons was more caudal to the level of the contralateral axons and could be observed in the same section as the EVN, at approximately −5.8 mm from Bregma (Figure 4G). We summarized the trajectory and the sparser density of ipsilaterally projecting EVN axons in a schematic drawing (Figure 4J). Starting from the EVN, ipsilaterally projecting fibers curved ventrally to avoid the genu of the 7th nerve (g7n) located medially, and the magnocellular and parvocellular MVN (MVNmc, MVNpc) located superiorly. The fine axon fibers (Figures 4H,I, arrowheads) were easily distinguishable from EVN dendrites (Figure 4I, arrows), which had characteristically thicker and arborized morphology. The ipsilaterally-projecting fibers traveled both dorsal and ventral to the inferior salivatory nucleus (IS), thus helping to delineate it (Figure 4H). After passing by the IS, the ipsilateral fibers reconverged to form a single bundle where they joined with other afferent and efferent vestibular fibers entering the vestibular root of the vestibulocochlear nerve (Figure 4; 8vn).

### The Peripheral Projections of the Efferent Vestibular System

To study the efferent innervation of the peripheral vestibular organs following intracranial injection of AAV, we used wholemounts of isolated, semi-intact, triad preparations comprising three conjoined vestibular organs: anterior crista (AC), horizontal crista (HC), and utricle (U; Figures 5a4,b1). We also used separate wholemounts of isolated posterior cristae (PC) and sacculus. This approach allowed us to preserve most of the vestibular neuroepithelium intact. However, due to the difficulty of isolation, saccular data remained incomplete. Nevertheless, data from the sacculus supported our general findings from the other vestibular organs.

In addition to being expressed in EVN cell bodies and dendrites, AAV-mediated and *ChAT-Cre*-dependent expression of eYFP and mCherry was observed in all peripheral vestibular organs (Figure 5). In most cases, endogenous expression was bright enough to observe efferent nerve fibers and bouton terminals within the peripheral vestibular organs, without immunolabeling enhancement (Figures 5B,D). Only the *septum cruciatum*, a characteristic non-sensory ridge in mouse vertical canals (AC and PC) was devoid of eYFP and mCherry fluorescence (AC: Figures 5b1,d1, arrows; PC: Figures 5b3,d3, arrows).

Vestibular organ wholemounts provided valuable information on the general presence of ipsi- and contralaterally-originating efferent projections. However, due to the curved surface of the vestibular cristae, capturing the detailed innervation patterns of efferent terminals is technically challenging. This is because observing a wholemounted cristae from above, for example, only provides a true indication of efferent terminal density where the surface of the crista is perpendicular to the line of sight. Viewing the remaining surface of the crista from above, particularly the sides of the crista, becomes increasingly problematic as the surface orientation, with respect to the line of sight, changes from perpendicular to parallel. Simply put, terminal density appears greater than it really is on the crista sides when viewed from above. This foreshortening effect is compounded in the cristae peripheral zones, where efferent terminals from both ipsi- and contralateral EVNs appeared to be more abundant when compared to central zones of the cristae (e.g., Figures 5b1,b2,d1,d2). This peripheral zone bias of crista efferent innervation was mirrored in the peripheral zones of the utricle (Figures 5b1,b2,d1,d2) and saccule (Supplementary Figures 1D–G). Therefore, for a more detailed examination, transverse sectioning of the vestibular organs was required (Figures 5b2,d2, dashed white lines). Immunofluorescent labeling was used to enhance eYFP and mCherry signal from fine terminal fields. As suggested by wholemounts, dense eYFP- and mCherry-positive efferent nerve fibers and boutons were found interspersed throughout the left and right AC (Figures 5c1–c3,e1–e3, arrowheads), however, peripheral regions were preferentially innervated (e.g., Figure 5c3). Similarly, eYFP- and mCherry-positive fibers and terminals were observed in the left and right HC along the whole length of the sections with a preferential peripheral distribution pattern (Figures 5c4–c6,e4–e6, arrowheads pointing at nerve end terminals). Similar interspersed distributions of ipsilateral and contralateral innervation were observed in the utricle (Figures 5c7–c9,e7–e9) and saccule (see Supplementary Figures 1D–G). Superimposing eYFP and mCherry images showed no differences in ipsilateral vs. contralateral efferent terminal fields within individual vestibular organs and although they were distributed throughout, in all vestibular organs: PC (Figures 5b4,d4), AC (Figures 5c3,e3), HC (Figures 5c6,e6), U (Figures 5c9,e9) there was a clear peripheral bias for both ipsi- and contralateral efferent innervation. All virally tagged fibers and terminals in the vestibular organs were positive for ChAT, and there were rarely ChAT-positive and eYFP- or mCherry-negative elements (data not shown). This confirmed our AAV labeling had captured the full complement of ChAT-positive efferent fibers and their associated terminal fields in the vestibular organs.

### Retrograde Tracing and Cell Counts of EVN Neurons

Our anterograde tracing studies confirmed our viral injection studies that suggested a larger proportion of EVN axons crossed the midline to innervate the contralateral vestibular organs (Figure 4), compared to ipsilateral axons. However, using anterograde tracing alone, we could not determine what proportion of the total population this represented. We, therefore, used *retrograde* Fluoro-Gold (FG) labeling of EVN neurons originating from unilateral injections into vestibular organs in the *ChAT-GCaMP6_f_* mouse strain to provide information on EVN laterality. This strain expressed the calcium indicator protein, GCaMP6_f_, in all cholinergic cells including the EVN (confirmed by ChAT counterstaining—results not shown). Since GCaMP6_f_ incorporates Green Fluorescent Protein (GFP) within its molecular structure, it can be visualized using standard GFP immunohistochemistry (Figures 6D–F). At the level of the EVN, this also included facial nerve genu (g7n), *abducens nucleus* (*abd*), and *facial nucleus* (*7n*; Figure 6D). Note, the EVN (Figures 6E,F), is clearly distinguished from the closest group of GFP-labeled cell bodies in the cholinergic abducens nucleus, located ventral to the facial nerve genu (Figure 6D).

Prior to the use of FG as a retrograde tracer, we injected Fast Green dye into the PC to confirm the spread from the PC to the rest of the vestibular portion of the membranous labyrinth (Figure 6A). Unilateral FG injections into the PC (Figure 6A, arrow) generated strong fluorescence in all ipsilateral vestibular neuroepithelia examined: AC, HC, U (Figure 6B), and the PC (Figure 6C). No FG label was observed in the ipsilateral cochlea, or contralateral labyrinth, or cochlear efferent neurons in the MOC and LOC of the superior olivary complex.

Immunofluorescent labeling was not required to amplify the FG label in peripheral or central tissue. However, GFP-immunolabeling was used to identify all GCaMP6/ChAT-positive EVN neurons. After a unilateral injection in the posterior canal, FG was retrogradely transported back to subsets of neurons in both left (ipsilateral) and right (contralateral) EVNs (Figures 6G arrows, H,J). While all FG-positive EVN neurons were double-labeled with GFP, not all GFP-positive EVN cells were double-labeled with FG (Figures 6J,K, arrows). Table 1 summarizes the results from four retrograde FG experiments. In all cases, there was a significant difference in the number of double-labeled (FG+GFP) cells between ipsilateral and contralateral EVNs. Based on the proportion of GFP+FG and GFP-only in EVN cells, approximately one-third (32%) of ipsilateral EVN neurons were retrogradely labeled with FG, whereas over half of contralateral EVN neurons (55%) were FG-labeled. It should be noted that in none of the eight EVNs retrogradely labeled with FG did we see any evidence for additional clusters as reported in other rodents such as chinchilla (Marco et al., 1993) or gerbils (Perachio and Kevetter, 1989).

**Table 1:** Ipsilateral and contralateral EVN neuronal cell counts of retrogradely labeled with Fluoro-Gold (FG) and expressing Green Fluorescent Proteins (GFP).

Animal ID	Relative to FG-Injection	GFP + FG Positive (%)	GFP Only (%)	Total (EVN cells)	2 × 2 Table, Fisher *p* value
#1	Ipsi	18 (36.7)	31 (63.3)	49	0.0159
	Contra	31 (62.0)	19 (38.0)	50
#2	Ipsi	17 (28.8)	42 (71.2)	59	0.0161
	Contra	31 (50.8)	30 (49.2)	61
#3	Ipsi	19 (34.5)	36 (65.5)	55	0.0193
	Contra	29 (58.0)	21 (42.0)	50
#4	Ipsi	16 (29.1)	39 (70.9)	55	0.0387
	Contra	23 (51.1)	22 (48.9)	45
**Average**	**Ipsi**	**17.5** ± 0.6* (32.1)	**37** ± **2.3** (67.9)	**54.5** ± 2.1*	**0.0200**
	**Contra**	**28.5** ± 1.9* (55.3)	**23** ± **2.4** (44.7)	**51.5** ± 3.5*

*Standard Error. Bold values represent averages.

Antibody labeling for GFP also enabled us to count the total number of EVN neurons. Table 1 summarizes the results. The average EVN cell number (± S.E.) was 53 ± 1.92 (ipsi EVN *n* = 4, average = 54.5 ± 2.1; contra EVN *n* = 4, average = 51.5 ± 3.5). There was no significant difference in the number of EVN neurons on opposite sides of the brainstem. The relatively small variation in the number of EVN neurons across the four animals and eight EVNs examined suggests a relatively stable cholinergic population. We also measured cell body cross-sectional areas, of all neurons in each of four EVNs, one EVN from each animal (Table 2). For a total of 210 EVN neurons measured, the average area (± S.D.) was 138.0 (± 38.2) μm^2^, with the unimodal distribution. The average longest axis was 17.1 (± 3.0) μm and the shortest was 11.2 (± 1.9) μm, which gives a roundness index of 0.6 (4*Area / Pi*Maximum diameter^2^), or oval appearance.

**Table 2:** Average (± S.D.) cell body cross-sectional areas and their maximum and minimum diameters of EVN neurons labeled with GFP/ChAT.

Animal ID	n	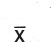 Area, μm^2^	Area, S.D.	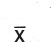 Max Dia, μm	Max Dia, S.D.	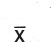 Min Dia, μm	Min Dia, S.D.
# 1	50	141.8	44.3	17.6	3.2	11.1	2.1
# 2	60	145.4	34.0	17.5	3.1	11.6	1.7
# 3	55	121.9	27.6	15.8	2.3	10.7	1.7
# 4	45	142.8	42.1	17.5	2.7	11.2	2.0
**Average**	**210**	**138.0**	**38.2**	**17.1**	**3.0**	**11.2**	**1.9**

Bold values represent averages.

## Discussion

### The Central Organization of EVN

The mammalian EVN was first described in kitten (Warr, 1975) using retrograde transport of horseradish peroxidase (HRP). Later tracing studies in rodent species, using additional labeling methods, identified the EVN in the guinea pig (Strutz, 1982), gerbil (Perachio and Kevetter, 1989), chinchilla (Marco et al., 1993; Lysakowski and Singer, 2000), and mouse (Leijon and Magnusson, 2014). Using transgenic mouse strains, we were able to anatomically target and characterize the mouse central and peripheral EVS to help fill important gaps in our understanding of this elusive sensory efferent circuit. Cre-dependent AAV-mediated expression of fluorescent reporters (Harris et al., 2012) provided highly specific labeling that allowed for visualization of neuronal cytoarchitecture and reliable tracing of axonal pathways to their terminal fields. Our retrograde FG data strongly supports a single EVN nucleus in mice with no other labeled clusters (cholinergic or non-cholinergic).

The clustering of EVN neurons suggests diversity among different mammalian species. In guinea pigs (Strutz, 1982) and gerbils (Perachio and Kevetter, 1989), two distinct clusters have been reported, and in chinchillas (Marco et al., 1993) and squirrel monkeys (Goldberg and Fernandez, 1980) there are putatively three. In mice, the EVN has been previously identified as comprising a single cluster of neurons, dorsolateral to the facial nerve genu (Leijon and Magnusson, 2014; Mathews et al., 2015), and is a location common to all mammalian species investigated to date, and often referred to as *group e*. Our results support these previous studies in mice. We found this single “central” cluster of densely packed multipolar cell bodies (see Supplementary Video), however, there were often examples of outlying or “peripheral” cell bodies, but still within 50 μm of the tightly packed core cluster. These outliers do not, in our opinion, constitute a separate EVN group, since there is no sharp delineation as seen in other larger mammals, which form two or more cell clusters. In addition, both core and outlying neurons were identified as cholinergic. This lack of extra clusters may be due to the smaller size of the mouse brain and fewer EVN neurons, when compared to their larger mammalian counterparts. However, it should be noted that in the much larger auditory efferent system, two different cell clusters, the lateral olivocochlear neurons (LOC) and the medial olivocochlear neurons (MOC; Guinan, 2006) have been retained in mice (see below, Anatomical comparison of the cochlear and vestibular efferent system).

It is possible the mouse EVN may form two clusters of neurons but have remained physically close so that they appear as a central “core” and peripheral outliers, forming one EVN. But any differences between the core and outliers have yet to be determined.

To expand on the potential role of multiple EVN cell clusters in the species mentioned above, it may be worth considering their habitat type and their relative brain sizes. Chinchillas live in rocky mountain areas, while squirrel monkeys live in the canopy of tropical rainforest (Miller et al., 1983; Boinski, 1987). In contrast, the ancestors of the domesticated guinea pig and the gerbil lived on open grassy plains (Zhou and Zhong, 1989; Cassini and Galante, 1992). The two former species are extremely agile, which presumably requires a well-developed locomotor and balance system. Whereas guinea pigs and gerbils move about at ground level. Unsurprisingly, the brain size of the squirrel monkey is the biggest among these species, about 23 g (Hartwig et al., 2011). However, the chinchilla brain is only slightly larger, 5.25 g compared to the guinea pig, 4.28 g. The brain sizes of the gerbil and mouse are 0.9 g and 0.5 g, respectively (Wilber and Gilchrist, 1965; Sacher and Staffeldt, 1974; Goffinet and Rakic, 2012). It is plausible that differences in habitat and lifestyle may result in differently developed vestibular systems and the number of EVN cell clusters may reflect this; one or two EVN clusters in the less agile species (mouse, gerbil, guinea pig) and three clusters in more agile species (chinchilla, squirrel monkey).

In mice, the EVN was found clustered around a characteristic blood vessel, similar to that reported in gerbils (Perachio and Kevetter, 1989). This makes the nucleus potentially vulnerable to increased blood pressure. In addition, the proximity to the circulatory system also provides means for EVN neurons to secrete neuromodulators directly into the bloodstream. This could have clinical implications given that one of the neuropeptides found in EVN neurons is CGRP (Perachio and Kevetter, 1989; Luebke et al., 2014; Jones et al., 2018) a neuroactive substance known to be implicated in migraines (Goadsby et al., 2017). Therefore, EVN neurons may be involved in vestibular migraines.

Fluorescent labeling of the extensive dendritic architecture of the EVN allowed us to follow distal projections towards neighboring brain regions, especially the medial vestibular nucleus (MVN). This suggests a reciprocal influence of the vestibular nucleus complex (VNC) on the function of EVN neurons. A close association between VNC and EVN is consistent with a report that described polysynaptic inputs onto the EVN after rabies viral tracing, which identified the VNC as one of the major contributors (Metts et al., 2006).

### Axonal Pathways From the EVN

In mice, individual EVN neurons project either to the ipsilateral or contralateral eighth nerve. We found no evidence for bilateral projections. We observed contralateral bound axons crossing the midline of the brainstem, approximately 100 μm more rostral to the level of the EVN neurons, suggesting a rostral-looping trajectory of the contralaterally projecting fibers before they turned back caudally to join ventrally projecting ipsilateral EVN fibers, as they exited towards the eighth nerve. Therefore, we did not observe any crossing axons in sections containing EVN neurons and this is the likely reason midline electrical stimulation of crossing efferent fibers was successful rostral to the known location of the EVN cell bodies (Schneider et al., 2021). This rostral decussation has also been reported in gerbils (Perachio and Kevetter, 1989) and is possibly the case in other mammalian species (Goldberg and Fernandez, 1980; Dechesne et al., 1984; Perachio and Kevetter, 1989). As they cross the midline, axons originating from left and right side EVN were intermingled and did not show any compartmentalization within the crossing “bundle”.

Ipsilateral fibers join the genu of the 8th cranial nerve (g8n), at the level of the EVN, more caudal to the level of the contralateral fibers. The visualization of ipsilateral fibers is more difficult for two reasons: (1) there are fewer ipsilateral axons than contralateral axons; and (2) ipsilateral axons do not overlap or intermingle with other fibers, and their density is significantly lower making it harder to visualize them. Somewhat surprisingly, ipsilateral fibers project ventrally past the genu of the 7th cranial nerve avoiding the magno- and parvocellular MVN, despite the shortest route to the 8th cranial nerve would be straight across the MVN.

We hypothesize that the trajectories of the contralateral and ipsilateral efferent vestibular tracts are significantly impacted by other members of the vestibular nuclear complex during development. Thus, the contralateral fibers were pushed more rostrally from the EVN, while the ipsilateral fibers project more ventrally.

Our retrograde labeling results are similar to those reported previously in other mouse studies (Leijon and Magnusson, 2014; Mathews et al., 2015). A subset of GCaMP6_f_ -positive EVN neurons, on both ipsi- and contralateral sides, were FG-labeled, the majority of which were found contralaterally. This is consistent with all previous mammalian studies and demonstrates significant contralateralization of the EVS. Based on the number of GFP/ChAT positive neurons that were either FG-positive or FG-negative on each side, we determined that, on average, EVN comprises over half (55.3%) contralaterally, and one third (32.1%), ipsilaterally projecting axons (Table 1). Our results were remarkably consistent despite the inherent variability of retrograde labeling. Indeed, Animal #1, which we consider as our best retrograde labeling example, a unilateral FG injection in the vestibular periphery accounted for 36.7% of ipsilateral EVN population and 62.0% contralateral EVN population. Taken together, these data account for 98.7% of a generic EVN nucleus, making bilateral projections from individual EVN neurons highly unlikely. While this finding is in agreement with other mammalian studies, including gerbil, it contrasts with a previous study in cat, that suggests up to 20% are bilaterally projecting (Dechesne et al., 1984). We attempted to confirm our result by injecting a second retrograde tracer, Fluoro Ruby (FR), into the contralateral posterior canal and look for double-labeled EVN neurons. However, we found transport from the inner ear using FR much less efficient and more variable when compared to FG, and therefore, FR results were not reported.

### Peripheral Projections of the EVN

**Figure 7:**
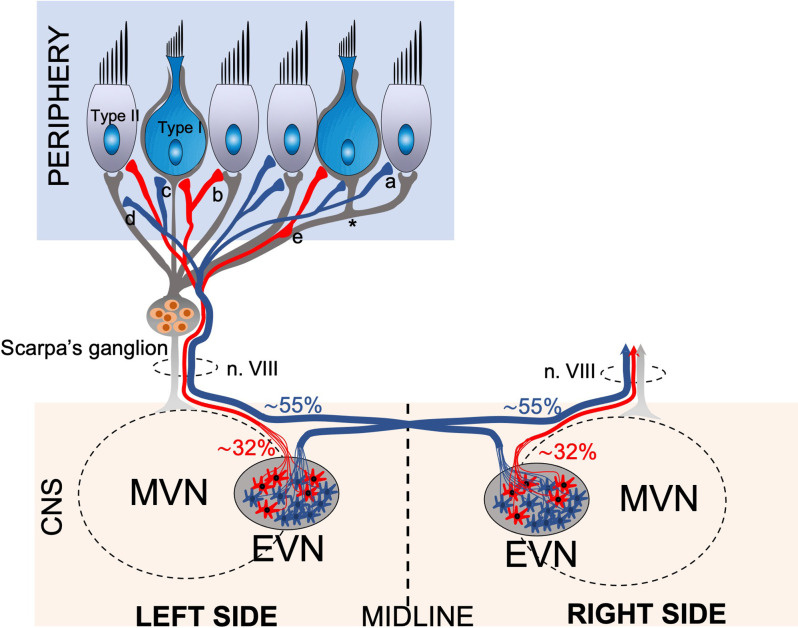
Schematic drawing of the anatomical projections of the Efferent Vestibular System. Schematic of efferent (red, blue) and afferent (gray) innervation of the vestibular neuroepithelium, which consists of amphora shape Type I and cylindrical shape Type II hair cells. The afferent fibers originating from the bipolar neurons of Scarpa’s ganglion transmit the vestibular sensory information from the labyrinths to the Vestibular Nuclei Complex (VNC) in the brainstem. The afferent fibers terminate in bouton terminals on Type II hair cells, calyx terminals on Type I hair cells, and dimorphic terminals comprising a bouton and a calyx (*). The efferent vestibular system originates from the Efferent Vestibular Nucleus (EVN) located in the brainstem. The EVN axons join the afferent fibers and travel in the vestibulocochlear nerve (n. VIII) to the neuroepithelium. Efferent fibers make synaptic contacts to the outer surface of type II hair cells **(a)**, afferent boutons **(b)**, calyx terminals **(c)**, afferent fibers **(d)**, and en-passant synapsis formed by axonal varicosities to afferent fibers **(e)**. Approximately 55% of EVN neurons provide contralateral (opposite side) projections, the axons cross the midline and join the opposite side vestibulocochlear nerve, while approx. 32% of neurons project ipsilaterally (same side).

Peripheral EVN projections to the vestibular organs in mice have not been as widely studied. To date, there are no detailed reports of EVN peripheral projections in mouse vestibular organs. Our data show that the vestibular neuroepithelia receive innervation from both ipsilateral and contralateral EVN, as reported previously in other mammals. This observation is consistent with the only two anatomical studies to date describing the efferent innervation pattern of the vestibular periphery using anterograde biocytin tracing in the gerbil (Purcell and Perachio, 1997) and radioautography in the cat (Dechesne et al., 1984). The small number of EVN neurons in mice (*n* = 53), means efferent axons branch extensively to make many direct contacts with afferent fibers, afferent terminals (calyces), and type II vestibular hair cells within the vestibular peripheral organs (Figure 7). For recent reviews, see Mathews et al. (2017), Poppi et al. (2020), and Cullen and Wei (2021). Overall, our AAV tracing study presents evidence that both central and peripheral zones of the mouse vestibular cristae receive mixed innervation from both the ipsi- and contralateral EVN, although the majority of efferent contacts are from the contralateral EVN. In all mouse vestibular organs investigated, there appears to be no distinctive ipsilateral or contralateral innervation pattern as described previously in gerbil (Purcell and Perachio, 1997), where ipsilateral terminals innervate the central or apex zone of the crista, while contralateral terminals innervate the peripheral and planum zones. However, we did observe peripheral and planum regions of the cristae are favored both by a greater density of efferent terminals, and more efferent axons tend to pierce the neuroepithelial basement membrane in peripheral regions. This general mixed innervation pattern with a peripheral preference was also present in the utricle and saccule.

### Factors to Consider

The most detailed study of efferent innervation of peripheral vestibular organs is in gerbils, and while our results in mice share many similarities, it is important to consider the differences. Gerbils and mice not only differ in size, but also in behavior, ecology, and taxonomy. It has been shown that habitat type, diet, zonation, and activity timing, all have an impact on brain size and development (Mace et al., 1981). In addition, molecular taxonomy suggests that although they are both rodents, the Gerbilinae family is more related to spiny mice (Acomys) than true mice (Murinae) (Chevret et al., 1993; Waiblinger, 2010), suggesting a different phylogenetic lineage. Perhaps the most obvious difference is the size of the gerbil brain, which is almost twice as big as the mouse, and this is reflected in our own results where the cross-sectional area of individual EVN neurons is almost twice as big in gerbils (259.8 ± 75.2 μm^2^), when compared to mice (138.0 ± 38.2 μm^2^). Gerbils have two recognized clusters of EVN cells, the bigger cell cluster of 200 neurons (*group e*, Purcell and Perachio, 1997) is thought to be equivalent to the single cluster mouse EVN of 53 neurons. In addition, there is a more ventral cell group which has no equivalent in mice. These differences suggest the function of the gerbil and mouse vestibular system may also differ due to interspecific behavioral and ecological differences and our morphological findings may reflect this.

### Anatomical Comparison of the Cochlear and Vestibular Efferent Systems in Mice

There are critical similarities and differences between the two inner ear efferent systems in mice that are directly related to our findings. The mouse cochlea, with a total of ~3,500 hair cells (765 IHCs and 2,625 OHCs; Ehret and Frankenreiter, 1977), receives efferent innervation from 475 superior olivary complex (SOC) neurons (Campbell and Henson, 1988). In mice, the five vestibular organs on one side, have a total of ~10,500 hair cells (Lim and Brichta, 2012), and receive innervation from approximately 53 EVN neurons. As means of comparison, a very crude index of auditory efferent “branching” would suggest a ratio of approximately one central efferent neuron for every seven cochlear hair cells. In contrast, the vestibular efferent branching index has one efferent neuron for every 210 vestibular hair cells. This suggests a greater than an order of magnitude more exuberant vestibular efferent branching. In addition, the ratio of vestibular afferent to efferent neurons is lower in mice than for those typically reported for larger mammals. With approximately 3,500 vestibular afferents (Bäurle and Guldin, 1998) and 53 efferent neurons, the ratio is approximately 70:1, whereas it has been reported there is a ratio of 20:1 in guinea pigs, chinchilla, and squirrel monkeys (Lysakowski and Goldberg, 1997; Holt et al., 2011; Ryugo et al., 2011).

There are two efferent cell groups within the SOC: (1) the lateral olivary complex (LOC), which provide 65% of the total efferent innervation to the cochlea on the same side; and (2) the medial olivary complex (MOC), which provides the remaining 35% of the efferent innervation to the cochlea but coming mostly (75%) from the contralateral MOC (Campbell and Henson, 1988; Brown, 1993). In contrast, there is only one group of EVN neurons on each side. Therefore, EVN laterality more closely resembles the MOC than the LOC. Cochlear targets of LOC and MOC efferent neurons are different. LOC neurons innervate cochlear afferent fibers and inner hair cells, while MOC neurons innervate outer hair cells. EVN terminals are distributed throughout the vestibular organs although their axons are preferentially distributed in peripheral rather than the central zones of vestibular neuroepithelia. EVN terminals target vestibular primary afferent parent axons and their calyx terminals, and only innervate type II hair cells, since type I hair cells are typically isolated from direct efferent contact due to the intervening/surrounding calyx terminal. Centrally, MOC and maybe LOC axons form collaterals to the cochlear nucleus on their way to the periphery (Brown, 1993). We were unable to verify the presence of EVN collaterals projecting to any vestibular nucleus on their way to the periphery. Nor could we confirm the collateral EVN projection to the cerebellar flocculus, ventral paraflocculus, and interstitial nucleus of the vestibular nerve as seen in the gerbil (Perachio and Kevetter, 1989; Shinder et al., 2001). It should be noted, however, that in addition to the EVN nucleus, in two cases, we found doublelabeled (yellow) cells in a discrete region of the cerebellar vermis (see Supplementary Figures 1A–C). How these cells were double-labeled remains unknown.

Vestibulocochlear efferents originate from the same pool as facial branchial motor neurons in the brainstem (Fritzsch and Elliott, 2017). Given their common developmental origin, the principal fast neurotransmitter of both vestibular and auditory efferent systems is acetylcholine (ACh). In addition, LOC efferents have been reported to contain γ-aminobutyric acid (GABA), calcitonin-gene-related peptide (CGRP), and dopamine (Eybalin, 1993). MOC efferents have also been reported to also contain GABA and CGRP (Maison et al., 2003). In the EVS, ACh, and CGRP colocalize and are likely the dominant neurochemicals (Luebke et al., 2014; Jones et al., 2018), but there are also reports about the presence of enkephalins (Ryan et al., 1991) and μ-opioid receptors (Popper et al., 2004), but the presence of GABA and nitric oxide in efferent terminals has not been confirmed.

## Conclusion

The function of the cochlear efferent system is far better understood than the efferent vestibular system. In the cochlea, the efferent system plays a role in unmasking the signal from noisy backgrounds, protection of acoustic trauma, interaural sensitivity by influencing the stiffness of the tectorial membrane and modulating cochlear sensitivity in response to visual or acoustic stimuli during selective attention (Délano Reyes and Elgoyhen, 2016). In contrast, the exact function of the efferent vestibular system is still unknown. Recent evidence in mice suggests there are both fast and slow excitation effects when the EVS is electrically activated (Cassini and Galante, 1992; Schneider et al., 2021). However, precisely when the EVS is naturally activated and under what conditions still remains to be determined. Two behavioral reports suggest the EVS is involved in longer term events such as adaptation and compensation of the vestibuloocular reflex (Hübner et al., 2015, 2017).

The anatomical data described in this study are in line with our physiological characterization of mouse vestibular afferent responses to efferent stimulation (Schneider et al., 2021) and also a study in chinchilla (Marlinski et al., 2004). First, midline stimulation, which only activates contralateral EVS neurons, excited most regularly- and irregularly discharging afferents consistent with deposition of contralateral efferent terminals throughout the vestibular neuroepithelia. Second, ipsilateral stimulation, which recruits both contralateral and ipsilateral EVS neurons, also excited most classes of afferents, but could be distinguished from contralateral stimulation in that the resulting excitation was significantly larger. Such excitation patterns reinforce the notion that ipsilateral efferent terminals are also broadly distributed across the neuroepithelia, and not representative of any discrete regional EVS innervation pattern as reported in gerbils (Purcell and Perachio, 1997).

In summary, our anatomical study contributes details about the central and peripheral organization of the mouse EVS and provides a context for comparison with the auditory efferent system. In addition, knowing the precise anatomical arrangement of the EVS allows us to better interpret existing studies examining the effects of electrical activation of the EVS in anesthetized mice. These anatomical data will also be important for developing future experiments in awake, behaving mice where the goal will be to record EVS activity in real-time. After more than 60 years since their discovery, results from the *in vivo* experiments described above will finally begin to reveal the role of the EVS in balance after a long and winding road of discovery.

## Data Availability Statement

The raw data supporting the conclusions of this article will be made available by the authors, without undue reservation.

## Ethics Statement

The animal study was reviewed and approved by Animal Care and Ethics Committee, The University of Newcastle, Callaghan, NSW 2308, Australia.

## Author Contributions

DL, AB, and LP designed the study. DL, LP, and HD conducted the experiments. DL, AB, LP, and RL analyzed the data and made the figures. DL, AB, JH, and LP drafted the manuscript. DL, AB, LP, RL, and JH edited the manuscript. All authors contributed to the article and approved the submitted version.

## Conflict of Interest

The authors declare that the research was conducted in the absence of any commercial or financial relationships that could be construed as a potential conflict of interest.

## Publisher’s Note

All claims expressed in this article are solely those of the authors and do not necessarily represent those of their affiliated organizations, or those of the publisher, the editors and the reviewers. Any product that may be evaluated in this article, or claim that may be made by its manufacturer, is not guaranteed or endorsed by the publisher.
