# Supplementary figures and images for: The Long and Winding Road—Vestibular Efferent Anatomy in Mice
Source: Front Neural Circuits. 2022 Jan 28;15:751850. doi: 10.3389/fncir.2021.751850 (PMC8832101; doi:10.3389/fncir.2021.751850)

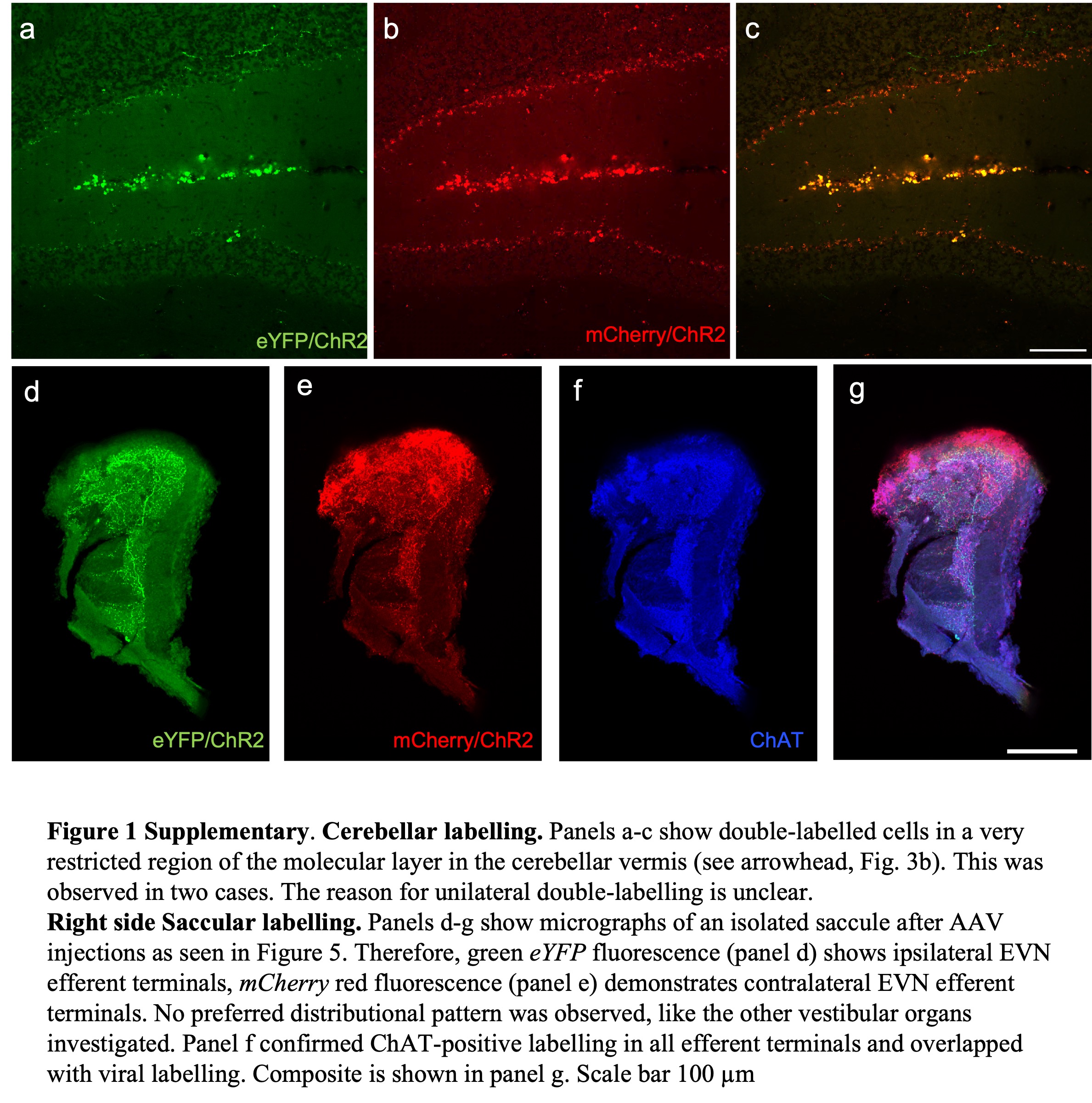

Supplement: Supplementary file 1 [file Image_1.jpg]
